# An Exploratory Qualitative Analysis of Explanations for COVID-19–Related Racial Disparities Among St. Louis Residents: “I Don’t Really Pay Attention to the Racial Stuff Very Much”

**DOI:** 10.5888/pcd20.230103

**Published:** 2023-11-09

**Authors:** Camille Kroll, Mikayla A. Johnson, Maura M. Kepper, Niko Verdecias, Matthew W. Kreuter

**Affiliations:** 1 Health Communication Research Laboratory, Washington University, St. Louis, Missouri; 2 Prevention Research Center, Washington University in St. Louis, St. Louis, Missouri; 3 College of Health Solutions, Arizona State University, Phoenix

## Abstract

**Introduction:**

Public health explanations for the disproportionate share of COVID-19–related illness and death among the Black population often differ from lay explanations, which can affect the public’s support for policies that address these disparities. This qualitative exploratory study examined the explanatory frameworks for COVID-19–related racial disparities in St. Louis among 54 St. Louis residents.

**Methods:**

From August 16, 2021, through May 20, 2022, we conducted semistructured interviews among a convenience sample of 54 St. Louis residents about their experiences during the COVID-19 pandemic. Directed content analysis identified participants’ explanatory frameworks for racial disparities in COVID-19–related illness and death. We disaggregated coded excerpts by race, age, education, and income to examine emerging themes.

**Results:**

Lay explanatory frameworks for racial disparities in COVID-19 included vaccine mistrust, lack of personal responsibility, low perceived susceptibility to COVID-19, pre-existing conditions or genetic predisposition, institutional racism, barriers to care, low socioeconomic status, insufficient information on COVID-19, and the inability to work remotely. Black interview participants addressed issues of systemic racism, inequitable allocation of COVID-19 vaccines, and institutional mistrust, whereas White participants did not directly acknowledge the role of racism. Both Black and White participants identified lack of personal responsibility among young Black people as a source of these disparities.

**Conclusion:**

This work identifies a need for improved health communication about racial disparities in COVID-19–related illness and death. Messaging that highlights racism may be less effective among the White population than the Black population in the US, whereas narratives that include the theme of individual choice may appeal broadly. Further research is needed on the use of communication strategies based on lay individuals’ explanatory frameworks for COVID-19–related racial disparities to enhance support for equitable public policy.

**Summary**

**What is already known on this topic?**

Public health and lay explanations for racial disparities in health outcomes often differ. This difference affects how people perceive their own health risks and their support for policies that address health disparities.

**What is added by this report?**

We examined the explanations that 54 St. Louis residents gave for COVID-19–related racial disparities in St. Louis. Although Black interview participants acknowledged the role of racism, White participants did not. Both groups emphasized personal responsibility.

**What are the implications for public health practice?**

Messaging that highlights racism may be less effective among the White population than the Black population in the US, whereas narratives that include the theme of individual choice may appeal to both.

## Introduction

Mirroring national trends, Black St. Louis residents have had disproportionate rates of illness and death from COVID-19 (1,2). The field of public health explains these racial disparities by implicating multiple adverse social determinants of health, including poverty, residential overcrowding, lack of health insurance, overrepresentation in the essential workforce, and systemic racism (3–5). The Black population in the US has also had lower rates of COVID-19 testing and vaccine uptake, although the reasons for these lower rates are not clear (5–10). Some studies indicate that vaccine hesitancy among the Black population is due to a well-founded mistrust of health care institutions predicated on contemporary and historical medical racism (6,11), a form of racism perpetuated by health care practices, teachings, and norms that include US government–sanctioned experiments on members of the Black population (eg, the Tuskegee Syphilis Study) and discriminatory patient–provider encounters. Other research has found no differences in willingness to participate in COVID-19 vaccination and testing between the White and Black population in the US (7), instead highlighting the inequitable rollout of COVID-19 vaccines, including in St. Louis (5,7–9). An overreliance on the role of mistrust in vaccine hesitancy potentially casts blame for lower vaccination rates on the Black population rather than on systemic issues of access and can further perpetuate medical racism.

Although US public health officials and the media have highlighted racial disparities in COVID-19–related illness and death, a growing body of evidence suggests that communicating about these disparities may have had the unintended consequence of making the White population feel less at risk than racial and ethnic minority populations for the severe outcomes of COVID-19 and less supportive of public health policies to address these disparities (12–14). This previous research focused more on educating the public on the existence of racial disparities in COVID-19 rather than examining the public’s own explanations for these disparities. These explanations may contribute to the extent to which audiences are receptive to public health messages. Public health organizations often emphasize the role of social determinants of health in creating racial disparities in health outcomes, but the concept may not be reflected in the lay population’s own explanatory frameworks (15). In this study, we define the lay population as people who do not have professional or academic knowledge of the field of public health. Literature on how the lay population explains health disparities demonstrates that the White population in the US often minimizes the role of racism and instead focuses on the effects of socioeconomic status, personal responsibility, and genetic vulnerability (15–21). The Black population in the US acknowledges the effect of racism but also asserts the role of individual choice (15,22,23).

The objective of this study was to identify lay explanations for racial disparities in COVID-19 illness and death in St. Louis and examine whether and how they differ by a person’s race, education, income, and age.

## Methods

This exploratory qualitative research used a portion of interviews from a larger mixed-methods cross-sectional study performed as part of the Health Communication Research Laboratory (HCRL) at Washington University in St. Louis’s participation in the Center for Disease Control and Prevention’s Prevention Research Center (PRC) Vaccine Confidence Network, which aims to build COVID-19 vaccine confidence and uptake in local communities (24). Eligibility requirements for participating in an interview included living in St. Louis or the St. Louis County metropolitan area at the time of the interview and being aged 18 years or older. Participants were recruited primarily through a convenience sample via distribution of a community-based flyer. We used this method of recruitment because it maximized the speed of data collection and the dissemination of data to local community partners during the rapidly changing pandemic environment. Institutional review board approval was obtained from Washington University in St. Louis.

From August 16, 2021, through May 20, 2022, two HCRL staff members conducted semistructured interviews with 54 participants about their experiences during the COVID-19 pandemic. The interview guide drew from the common data elements of the PRC Vaccine Confidence Network. Each interview lasted approximately 30 minutes and was conducted via Zoom (audio only). One interviewer identified as a young Black female and the other as an older White male. Interviews covered various topics, including the personal effect of COVID-19, the effect of the pandemic on the local community, perceptions and concerns about COVID-19 and the vaccine, and COVID-19–related racial disparities in St. Louis. We collected data on each participant’s demographic characteristics (age, sex, race and ethnicity, education level, and income level) and asked questions about their COVID-19 vaccination status. We also asked whether they or someone close to them had contracted COVID-19 and, if so, whether that person had been hospitalized or died. Each participant received a $50 Target gift card for their time.

The 54 audio-recorded interviews were professionally transcribed verbatim. One coder (C.K., who identifies as a young White female) performed directed content analysis on the transcripts (25,26) and examined the response to the question, “In St. Louis, Blacks have been affected more by COVID than other groups. Why do you think that is?” The codebook included the a priori code “Impact_Blacks” to identify the relevant interview sections and emerging subcodes relating to explanations for racial disparities in COVID-19. The coder (C.K.) performed 3 rounds of coding and updated the codebook after each round. After the second round of coding, the codebook was discussed by the initial coder and 2 members of the HCRL team (M.A.J. and M.M.K.) who had previously coded the data for other internal research reports. The final round of coding resolved any inconsistencies and grouped findings into themes. Representative quotes were extracted for each theme. All coding was performed in NVivo 20 (QSR International). An audit trail was created with detailed notes throughout the coding process. Data saturation was achieved. 

After the 3 rounds of coding were completed, the coded interview excerpts were disaggregated by demographic characteristic: race (non-Hispanic Black [hereinafter, Black], non-Hispanic White [hereinafter, White], “Other,” or refused); education level (less than college, some college or 4-year degree, or other advanced degree); annual household income (<$25,000, $25,000 to <$75,000, ≥$75,000, or refused); and age (19–45 or >45 years, with groups based on a histogram analysis of age distribution). For the purposes of this study, study participants who identified as Asian, Hispanic or Latino, or an unspecified race or ethnicity were grouped into “Other” because differences and similarities in explanations for COVID-19 racial disparities between Black and White participants were of primary interest. We also examined potential themes by education, income, and age among participants.

## Results

Most (61.1%) participants were non-Hispanic Black and most (81.5%) were women. Overall, 49 (90.7%) were vaccinated. Of the 5 unvaccinated participants, 3 were non-Hispanic Black and 2 identified as “other” race and ethnicity. The mean age was 46.8 years, ranging from 21 to 73 years. More Black participants (n = 17) than White participants (n = 4) indicated knowing someone in their social network who had been hospitalized or died as a result of COVID-19 (Table 1). Explanations for COVID-19–related racial disparities in St. Louis highlighted both individual and systemic factors (Table 2), and the 2 factors were often related (eg, personal mistrust in the COVID-19 vaccine and systemic racism). Individual factors included mistrust of the COVID-19 vaccine because of past government experiments on the Black population or conspiracy theories, lack of personal responsibility (particularly among young people in the Black community), low perceived susceptibility to COVID-19, and increased risk due to pre-existing conditions or genetic predisposition. Systemic factors encompassed institutional racism (eg, medical racism); barriers to COVID-19 care (eg, lack of transportation or health insurance); low socioeconomic status; insufficient information on COVID-19; and the inability to work remotely. Some participants (n = 8) denied any knowledge of these racial disparities and refused to speculate.

**Table 1:** Key Demographic Characteristics of Participants (N = 54) in Interviews About Reasons for COVID-19–Related Racial Disparities, St. Louis, Missouri, August 2021–May 2022^a^

Characteristic	No. (%)^b^
**Age, mean (range)**	46.8 (21–73)
**Sex**	
Female	44 (81.5)
Male	10 (18.5)
**Race and ethnicity**	
Non-Hispanic Black	33 (61.1)
Non-Hispanic White	14 (25.9)
Other^c^	7 (13.0)
**Education level**	
Less than college	16 (29.6)
Some college or 4-year degree	30 (55.6)
Other advanced degree	8 (14.8)
**Household income in 2020, $**	
<25,000	13 (24.1)
25,000–74,999	26 (48.1)
≥75,000	11 (20.4)
Refused	4 (7.4)
**COVID-19 vaccination status**	
Yes	49 (90.7)
No	5 (9.3)
**Unvaccinated status, by race**	
Non-Hispanic Black	3 of 5 (60.0)
Non-Hispanic White	0
Other	2 of 5 (40.0)
**Someone in social network hospitalized or died as a result of COVID-19, by race**	
Non-Hispanic Black	17 of 33 (51.5)
Non-Hispanic White	4 of 14 (28.6)
Other	3 of 7 (42.9)

a From August 16, 2021, to May 20, 2022, semistructured interviews were conducted with a convenience sample of 54 St. Louis residents about their experiences during the COVID-19 pandemic.

b Values are number (percentage), except for age. Denominator for all percentages is 54, unless otherwise indicated.

c For the purposes of this study, study participants who identified as Asian, Hispanic or Latino, or an unspecified race or ethnicity were grouped into “Other” because differences and similarities in explanations for COVID-19 racial disparities between Black and White participants were of primary interest.

**Table 2:** Explanations for COVID-19–Related Racial Disparities Identified by Interview Participants (N = 54), St. Louis, Missouri, August 2021–May 2022^a^

Theme	Representative quotes	Race and age, y, of participant
**Individual factors**		
Vaccine mistrust as product of history of government experimentation on Black people	African Americans have a history, as you well know, not only of past discrimination but discrimination up until this day to the point where we’ve been experimented on with different medications and things like that. First, it was a lot of people, Black people, bein’ affected because they didn’t trust the vaccine.	Black female, 64
	When they were giving those Black men in Africa syphilis, to see how fast they would die. They told them it was something for something else. We know, we remember. We don’t need to have all the information. We just know that something happened. Then it involve White guys and needles.	Black male, 35
Vaccine mistrust as product of conspiracies or misinformation	Because they are listening to conspiracy theories…Well, one’s the conspiracy is Bill Gates. . . . Yeah, Bill Gates is a hit man for government.	Black female, 72
	I think maybe misinformation that already preys on the mistrust that exists in the city.	White female, 26
Lack of personal responsibility	They don’t want to take the vaccine, and they’re not staying in. They can’t be confined in the home. . . . They’re used to going out doing what they’re doing, and they just don’t listen.	Black female, 67
	A lot of the younger people — when I say younger, I’m gonna say 35 and under. They don’t know how to sit in the house. What I mean by that is, if you tell us that, ‘Oh, you gotta stay in the house,’ we're gonna stay for a certain amount of time. Then it’s like oh, I’ma take a chance. Next thing you know, you start seeing parties.	Black male, 35
Lack of perceived risk	They don’t believe what the numbers are telling them. Young people don’t believe that it can affect them. They don’t believe they can get COVID or give it to their relatives.	Black female, 41
	I think a lot of it is because you got people that does not take stuff serious. They take things for granted. I think a lot of people didn’t think that COVID was as bad as it was.	Black female, 65
Increased risk due to pre-existing conditions or genetic predisposition	Because we [Black people] have more existing conditions probably than any other race.	Black male, 35
	Well, it seems to me, I got the impression that they [Black people] had a genetic predisposition.	White female, 56
**Systemic factors**		
Institutional racism (eg, medical racism) and resulting mistrust	When you’re Black, you naturally have a distrust for the medical community.	Black male, 35
	I would assume it has to do with just the general distrust in health care or the health system, given some not-so-good history there.	White female, 35
Barriers to COVID-19 care (eg, availability of transportation, access to vaccines or testing, health insurance)	One, because a lot of us don’t have insurance.	Black female, 64
	They [Black people] wanted the vaccinations as soon as they came [per a Black colleague]. I really don’t know if it’s because they didn’t have the same access.	White female, 56
Low socioeconomic status	Because of the economic despair in the Black community, they fall victim to COVID a lot easier and a lot quicker.	Black male, 54
	I do think it has a lot to do with the socioeconomic divide here in St. Louis. . . . Along with many White people, a lot of African Americans are in that lower end as well.	White female, 35
Lack of information on COVID-19	Probably because they [Black residents] don’t get the same information as we do.	White female, 61
	Also, because a lack of education or information that the people needed to go farther to get help. Because they didn’t have the – they wasn’t educated on the vaccines and educated on what could happen, a lot of ‘em just felt like they’d just take their chances.	Black female, 65
Inability to work remotely	A lotta the people that we – that I function with and serve are people who have some of the frontline jobs. Meaning, they’re grocery store clerks, or they’re janitors, or they’re people who actually had to keep working all through the pandemic.	White female, 63
	We were the ones on the front lines. We were the ones that were working at the McDonald’s, at the hotels, so more of us were working there and more of us were getting sick because we were on the front line other than the nurses. We were in the restaurants. We were in the hotel industries. We were in those minimum wage jobs.	Black female, 59
**Other**		
Lack of knowledge of racial disparities	I actually wasn’t aware of that. . . . I don’t really pay attention to the racial stuff very much.	White female, 36
	Oh gosh, I don’t know. I really don’t know. I have no knowledge of that.	White female, 60

a From August 16, 2021, to May 20, 2022, semistructured interviews were conducted with a convenience sample of 54 St. Louis residents about their experiences during the COVID-19 pandemic.

The most notable differences in lay explanations for COVID-19–related racial disparities in St. Louis occurred along racial lines rather than by education, income, or age. We found no major differences across unique combinations of demographic characteristics (eg, Black females with less than a college education), perhaps because we did not achieve data saturation among small subgroups.

### Black participants

Black participants emphasized the historical role of the US government in perpetuating racism and the contemporary role of medical racism in vaccine hesitancy and mistrust of government and hospitals among Black St. Louis residents. They frequently mentioned government experimentation and the Tuskegee syphilis study, with 1 participant stating, “There’s just been a bad history of experimentation and stuff. Black people don’t trust doctors in hospitals” (Black female, aged 43). Unlike White participants, Black participants directly identified the role of medical racism and discrimination in their mistrust of the COVID-19 vaccine and hospitals: “We always the most affected, I guess because we’re the most experimented on. Then medical racism is a real thing because you can go to the doctor and be in pain and tellin’ them that you’re in pain but they’ll think you just lyin’” (Black female, aged 48).

Black participants also highlighted barriers among Black St. Louis residents to being healthy or receiving medical care, including poverty and lack of health insurance. One participant identified residential overcrowding as increasing the risk for COVID-19: “Their economic situation. A lotta African Americans are forced to cohabitate together, so you can have 10 people in a 2-bedroom home” (Black female, aged 64). Black participants also noted the effect of delayed access to information about the COVID-19 pandemic and lack of vaccine prioritization, despite the disproportionate share of COVID-19–related illness and death in the Black population: “They weren’t given priority with the vaccine even though the majority of us were dying and more of us were dying than any other population” (Black female, aged 59).

On a more individual level, Black participants frequently referred to the lack of perceived susceptibility to, and severity of, the disease among the Black population, which they implied may have led to riskier behaviors and more frequent infections compared with other racial and ethnic groups. Several participants noted that some Black people did not think they could contract COVID-19, although it is unclear from the interviews why they believed this: “Some of the Black people that I know said — would think that — that it was only White people was getting it and all that, ‘Well, we don’t get that kinda stuff’” (Black male, aged 54). Another participant expressed the idea that young people in her community were not worried about the potential severity of COVID-19: “Over here with our young people, the message was not connecting well, and they were told, it’s not going to impact you. It’s more the senior population” (Black female, aged 53).

### White participants

Although White participants noted the potential role of poverty in COVID-19–related racial disparities in St. Louis, they never directly mentioned the words “racism” or “discrimination.” Instead, they vaguely alluded to these concepts as primarily historical rather than contemporary phenomena: for example, a “horrible history” (White female, aged 63), “long and terrible story” (White female, aged 73), “not-so-good history” (White female, aged 35).

Several White participants asserted that Black people are more likely to get COVID-19 either because of “genetic predisposition” to the virus itself (White female, aged 56) or poor health from living in their communities. One participant explained, “I would say it’s health in general because a lot of African Americans are not healthy. They live in blighted communities” (White female, aged 61).

More White participants than Black participants denied any knowledge of COVID-19–related racial disparities in St. Louis, with one respondent stating, “I don’t really pay attention to the racial stuff very much” (White female, aged 36). When asked about her own race, she reported that she is “unfortunately . . . Caucasian,” seeming to potentially indicate her discomfort in talking about the topic.

### Similarities between Black and White participants

Both Black and White participants often referred to a lack of personal responsibility, particularly among young Black people, as causing COVID-19–related racial disparities in St. Louis. This idea often, although not always, was expressed by participants aged 30 years or older who were speaking about people younger than themselves. One woman explained, “Maybe because we’re [Black people are] not as cautious, could be, because people are still going out partying, the younger generation, partying in clubs, not wearing masks, they don’t wear masks in the store” (Black female, aged 67). Although some participants attributed this behavior among young people to their perceived lack of susceptibility to COVID-19, others viewed it as a lack of regard for anyone other than themselves: “Because [young people] do whatever they want and don’t care. They don’t wear masks. . . . A lot of those and the youngsters out here have no guidance. None, they aren’t just bad mistakes for that because they don’t give a f—, sorry, they don’t care, they get what they want” (White female, aged 34).

## Discussion

This exploratory qualitative study of St. Louis residents’ explanations for COVID-19–related racial disparities found that Black study participants often attributed these disparities to systemic racism and its role in vaccine hesitancy, misallocation of COVID-19 vaccines, and general mistrust of governmental and medical institutions. Notably, White participants never directly mentioned “racism” or “discrimination,” instead obliquely referencing injustices against Black Americans as a past (rather than continued) reason for disparities. Both Black and White participants noted a lack of personal responsibility, particularly among young Black people, as a factor contributing to COVID-19’s racial disparities in St. Louis. However, only Black participants addressed the issue of perceived susceptibility and severity, with several stating that they thought Black people, especially young ones, believed they could not get COVID-19, get very sick from it, or transmit it. These ideas somewhat mitigate the personal responsibility explanatory framework that otherwise implies a selfish and willful disregard for community health. Finding scapegoats during public health crises is common, and one group who often faces blame are those viewed as not following public health precautions, such as young people (27).

Our study results mirror previous research on differences between how White and Black Americans explain health disparities. That a White participant endorsed a genetic explanation for COVID-19’s racial disparities is consistent with previous literature (16,19). Our study also supports previous research that White Americans recognize poverty as a contributing factor more than racism (20,21). This viewpoint reflects White participants’ failure to address contemporary racism and discrimination, instead acknowledging neighborhood-level factors, such as poverty, independently of the structural racism at the root of segregated neighborhoods and poor health outcomes. Notable is one participant’s use of the phrase "blighted communities.” The term “blight” has a racist history in urban planning: largely low-income Black communities were declared “blighted” as a means of driving those populations out through various development projects (28). Declaring Black communities as “blighted” (28) and beyond repair without external remediation naturalizes disease within these communities and justifies allegedly race-neutral “cures” that primarily protect adjacent White middle and upper-class property values by preventing spread of the “blight” (28).

Aligning with previous studies (15–21), the role of personal responsibility and individual choice emerged as a key explanatory framework for COVID-19–related racial disparities among both Black and White study participants. Although Black Americans may acknowledge the importance of both personal responsibility and racism, researchers have found that it is difficult for many White Americans to disrupt their belief in the myth that contemporary society is just (29). When forced to examine their own privilege, individuals often uplift ideas of personal merit, rendering systemic inequities invisible (30).

Lay and public health explanations for COVID-19’s racial disparities did overlap in some areas, including how poverty, barriers to COVID-19 care, vaccine hesitancy, and overrepresentation in the frontline workforce contributed (3). We also found important differences between the study participants’ explanations and public health explanations, particularly interviewees’ emphasis on personal responsibility. This emphasis potentially moralizes the issue and casts blame on those who contract COVID-19. Framing health as a strictly personal endeavor helps to mask deeper systemic inequities and prevent their disruption.

It is important to garner public support for policies to reduce racial disparities not only in COVID-19 outcomes but also for other conditions that disproportionately affect racial and ethnic minority populations. Individuals’ explanatory frameworks for illness influence whether they view poor health as a personal failing, societal responsibility, or both. Our study suggests that White Americans may be less receptive than Black Americans to acknowledging racism as an underlying cause of health disparities. Communication strategies for the social determinants of health similarly recommend against framing health disparities as a primarily racial issue to avoid fostering negative bias from White participants (15). Both Black and White participants often viewed infection with COVID-19 as more of an individual choice than a systemic inevitability. Narratives that emphasize providing everyone the equal opportunity to exercise personal responsibility over their own health might be more broadly accepted, although the potential for this rhetoric to further entrench an individualism blind to systemic issues should be acknowledged (15). Randomized control trials might offer study participants various messages and then evaluate their receptiveness to those communication strategies based on their own explanatory frameworks.

### Strengths and limitations

Our study helps fill the absence in the literature of lay explanations for racial disparities in COVID-19. It is the only study to date that specifically explores how Black and White Americans explain COVID-19–related racial disparities. Although other studies assessed the association between participant education on racial disparities in COVID-19 and individuals’ consequent support of government intervention, none have examined people’s explanatory frameworks for these disparities (12–14). Some of these studies have incorporated measures of racial prejudice (12,14), but without examining participants’ personal explanations for racial disparities, it is difficult to design communication campaigns to enhance support for addressing these disparities. The qualitative nature of this study also elevates the lived experiences of St. Louis residents and demonstrates that lay explanations for poor health are often removed from public health explanations.

Our study has several limitations. Participants were selected as a convenience sample and selection bias likely affected who chose to be interviewed, including the fact that most of the sample had been vaccinated. The primary goal of qualitative research, however, is not generalizability, and the rich insight into local contexts outweighs these drawbacks. It is also difficult to tell whether White participants’ relatively limited knowledge of COVID-19’s racial disparities was true ignorance or a hesitancy to answer a potentially sensitive question. The 2 interviewers had distinctly different positionalities (a young Black female and an older White male) that could have affected their conversations with participants, although the Zoom calls did not use video. Although the primary coder’s positionality as a White woman inevitably affected her analysis of the data, the racial and ethnic diversity of the larger HCRL team contributed to creating an unbiased interpretation.

### Conclusion

Our study found that Black and White St. Louis residents have different explanations for COVID-19’s racial disparities in their city. Although Black participants acknowledged the role of racism, White participants did not. Both groups underscored the importance of personal responsibility. Future research should build the evidence base for better tailored messaging about racial disparities in COVID-19 and other health outcomes. Without understanding how people make sense of health disparities, public health risks 2 potentially harmful outcomes: wasting resources on ineffective messaging or, worse, further entrenching individuals’ negative attitudes toward specific groups of people or support for government intervention.
